# *N*-Methylation of Legionaminic Acid in *Legionella pneumophila* Serogroup 1 LPS Affects Cell Surface Properties, Intracellular Growth, and Cytokine Response

**DOI:** 10.3390/pathogens15060615

**Published:** 2026-06-09

**Authors:** Bożena Kowalczyk, Agnieszka Szuster-Ciesielska, Rafal Luchowski, Wiesław I. Gruszecki, Piotr Koper, Markus Petzold, Jacek Tarasiuk, Sofia Barigelli, Ermanno Federici, Marta Palusińska-Szysz

**Affiliations:** 1 Department of Genetics and Microbiology, Institute of Biological Sciences, Faculty of Biology and Biotechnology, Maria Curie-Skłodowska University, Akademicka 19 St., 20-033 Lublin, Poland; piotr.koper@mail.umcs.pl (P.K.); jacek.tarasiuk@mail.umcs.pl (J.T.); marta.palusinska-szysz@mail.umcs.pl (M.P.-S.); 2 Department of Virology and Immunology, Institute of Biological Sciences, Faculty of Biology and Biotechnology, Maria Curie-Skłodowska University, Akademicka 19 St., 20-033 Lublin, Poland; agnieszka.szuster-ciesielska@mail.umcs.pl; 3 Department of Biophysics, Institute of Physics, Faculty of Mathematics, Physics and Computer Science, Maria Curie-Skłodowska University, Radziszewskiego 10 St., 20-031 Lublin, Poland; rafal.luchowski@mail.umcs.pl (R.L.); wieslaw.gruszecki@mail.umcs.pl (W.I.G.); 4 Institute of Medical Microbiology and Virology, University Hospital Carl Gustav Carus, University of Technology Dresden, 01069 Dresden, Germany; markus.petzold@ukdd.de; 5 Laboratory of Applied and Environmental Microbiology, Department of Chemistry, Biology and Biotechnology, University of Perugia, 06122 Perugia, Italy; sofia.barigelli@dottorandi.unipg.it (S.B.); ermanno.federici@unipg.it (E.F.)

**Keywords:** *Legionella pneumophila*, lipopolysaccharide, legionaminic acid, TNF-α

## Abstract

*Legionella pneumophila* is widely recognized as the principal organism responsible for Legionnaires’ disease. Modifications of surface-exposed lipopolysaccharide in *L. pneumophila* are key determinants of bacterial adaptation to host cells. Using a mutant strain deficient in *N*-methylation of legionaminic acid and fluorescence-based biophysical analyses, it was demonstrated that *N*-methyl groups linked to the acetimidoylamino group of legionaminic acid modulate the physicochemical properties of the bacterial cell surface and influence the interaction of *L. pneumophila* with macrophages. Loss of *N*-methyl groups reduced the efficiency of bacterial interaction with THP-1-derived macrophages and impaired intracellular proliferation. In addition, the presence of *N*-methyl groups in legionaminic acid contributes to increased TNF-α production in THP-1 macrophages stimulated with *L. pneumophila* sg1 LPS, without affecting IL-6 induction, suggesting that *N*-methylation of legionaminic acid may skew the early pro-inflammatory response toward TNF-α-dominated signaling during *L. pneumophila* infection.

## 1. Introduction

*Legionella pneumophila* is a facultative intracellular, Gram-negative bacterium and the causative agent of Legionnaires’ disease [1]. In recent decades, a steady global increase in the incidence of *Legionella*-associated infections has been observed, with *L. pneumophila* serogroup 1 (sg1) accounting for approximately 80% of clinically confirmed cases [2,3]. In its natural aquatic environment, *L. pneumophila* predominantly replicates within free-living protozoa, including amoebae, which provide a protective intracellular niche and impose strong selective pressure on bacterial survival strategies. Mechanisms acquired during intracellular growth within protozoan hosts are subsequently exploited to infect and replicate within human macrophages, which constitute the primary cellular niche during the early stages of pulmonary infection [4]. Successful persistence within macrophages requires precise modulation of host innate immune signalling, allowing the bacterium to evade bactericidal responses while maintaining conditions favourable for intracellular replication [4].

A pivotal element of this host–pathogen interaction is lipopolysaccharide (LPS), the major surface-exposed component of the outer membrane of Gram-negative bacteria. In *L. pneumophila*, LPS not only contributes to membrane integrity but also plays a critical role in immune evasion and intracellular survival [5]. Structurally, LPS is composed of three domains: lipid A, a core oligosaccharide, and an O-specific polysaccharide (OPS). The lipid A moiety of *L. pneumophila* exhibits several unusual features, including a phosphorylated disaccharide backbone built of 2,3-diamino-2,3-dideoxy-D-glucopyranose (GlcN3N) residues, substituted with exceptionally long-chain and branched fatty acids such as 27-oxo-octacosanoic acid and heptacosane-1,27-dioic acid [6]. The presence of long-chain fatty acids in the lipid A of *L. pneumophila* is responsible for its significantly lower endotoxic activity compared with the highly toxic lipid A of *Salmonella enterica* serovar Minnesota [7]. The lipid A domain is linked to the inner core via a ketosidic bond between the C2 atom of a Kdo (3-deoxy-d-manno-2-octulosonic acid) residue and the C6 atom of the non-reducing GlcN3N unit. The inner core consists of two Kdo residues joined by a 2→4 ketosidic linkage, forming the structure α-Kdo-(2→4)-α-Kdo-(2→6) [8]. The outer core region is composed of an oligosaccharide enriched in 6-deoxy sugars and *N*-acetylated amino sugars, including rhamnose (Rha), mannose (Man), acetylquinovosamine (QuiNAc), and *N*-acetylglucosamine (GlcNAc), conferring a pronounced hydrophobic character to this region [9]. In *L. pneumophila* sg1, Philadelphia strain, the OPS consists of a homopolymeric chain with (2→4) linkages composed of a 5-*N*-acetimidoyl-7-*N*-acetyl derivative of 5,7-diamino-3,5,7,9-tetradeoxynon-2-ulosonic acid, commonly referred to as legionaminic acid [10]. The lack of free polar hydroxyl groups, together with deoxy substitutions at positions 3 and 9 of legionaminic acid, renders this homopolymer highly hydrophobic [7].

Genetic determinants of LPS biosynthesis in *L. pneumophila* sg1 comprise two principal regions: a highly conserved ~15 kb locus and a variable ~18 kb serogroup-specific region, partially disrupted by phage-related sequences. The latter encompasses genes implicated in late-stage OPS modification, including the ORF6–ORF11 cluster, which is unique to sg1 strains and is thought to contribute to serogroup-specific LPS architecture [11].

Within the species *L. pneumophila*, extensive genomic and functional heterogeneity among strains is observed, facilitating the selection of clones better adapted to infect the human host [12]. Among these adaptive traits is a phenotype characterized by resistance to complement-mediated killing in human serum, reduced susceptibility to neutrophil phagocytosis, and enhanced in vivo survival. This phenotype has been associated with the *O*-acetylation of the O-specific polysaccharide of *L. pneumophila* LPS, which is mediated by an *O*-acetyltransferase encoded by the *lag-1* gene [13]. Resistance to human complement coincides with resistance to bacteriophage LME-1 (*Legionella* mobile element–1) attachment, suggesting that environmental selective pressure may have driven the acquisition and persistence of this clinically relevant trait [14].

These observations indicate that structural modifications of LPS contribute substantially to bacterial fitness during infection and may represent attractive targets for novel therapeutic strategies. However, the specific contribution of *N*-methylation of legionaminic acid in LPS of *L. pneumophila* 130b strain to host–cell interactions has not been addressed. The study aimed to determine whether the *N*-methyl groups of the legionaminic acid present in the LPS of *L. pneumophila* are associated with the physicochemical properties of the bacterial cell surface, its ability to interact with THP-1-derived macrophages, and the induction of pro-inflammatory cytokines (IL-6 and TNF-α). To achieve these objectives, comparative analyses of the *L. pneumophila* wild-type strain and a mutant defective in legionaminic acid *N*-methylation were performed using physicochemical, FLIM-FRET, and immunological approaches.

## 2. Materials and Methods

### 2.1. Bacterial Strains

The study used *L. pneumophila*, clinical strain AA100/130b (ATCC BAA-74), the mutant-type strain 16D12 (LPS-A3), the complement of the mutant strain 16D12 named ΔORF8+pJB-ORF8, and control strains 130b+pJB1806, 16D12(ΔORF8)+pJB1806.

### 2.2. Construction of Mutant, Complemented, and Control Strains

The *L. pneumophila* mutant strain 16D12, carrying a disruption in the *orf8* gene within the LPS biosynthesis locus, was generated by random mini-Tn10 transposon mutagenesis, as described previously [15].

For complementation, a DNA fragment encompassing the LPW_RS04255 gene (ORF8) together with 200 bp upstream of the start codon was amplified from *L. pneumophila* 130b genomic DNA and cloned into the broad-host-range plasmid pJB1806, based on the RSF1010 replicon [16].

The resulting construct (ΔORF8+pJB-ORF8) was introduced into the 16D12 mutant by electroporation. Electroporation was performed using 0.2 cm gap electroporation cuvettes and a BTX ECM electroporator. Bacterial cells grown to the post-exponential phase (OD600 = 1.8) were washed three times with ice-cold wash solution containing 2% sucrose and 20 mM MgCl_2_, followed by one wash with 2% sucrose. Cells were finally resuspended in 2% sucrose, and 100 µL aliquots were mixed with plasmid DNA before electroporation. Electroporation was carried out at 2.3 kV, 200 Ω, and 0.25 µF. Immediately after pulsing, 2 mL of ACES-buffered yeast extract broth was added, and cells were allowed to recover for 16–20 h at 37 °C with shaking. Transformants were selected on BCYE agar supplemented with chloramphenicol (5 µg/mL), and plasmid presence was verified by PCR using gene-specific or vector-specific primers.

To control for potential effects associated with plasmid carriage, the empty pJB1806 vector was introduced into both the wild-type strain 130b and the mutant strain 16D12 using the same electroporation protocol. Transformants were selected on BCYE agar supplemented with chloramphenicol (5 µg/mL), and plasmid presence was verified by PCR using vector-specific primers.

### 2.3. Bacterial Culture

*L. pneumophila* strains were grown for 3 days on *N*-(2-acetamido)-2-amino-ethanesulfonic acid (ACES)-buffered charcoal-yeast (BCYE) extract agar, at 37 °C. The complement of the mutant strain 16D12(ΔORF8+pJB-ORF8), 130b+pJB1806 strain, and 16D12(ΔORF8)+pJB1806 strain were grown on BCYE medium containing chloramphenicol at 5 µg/mL. Bacterial biomass of *L. pneumophila* strains 130b, 16D12(ΔORF8), and 16D12(ΔORF8)+pJB-ORF8 harvested from agar plates was suspended in 0.5 M NaCl and centrifuged at 8000× *g* for 20 min. The pellet was subsequently washed with 0.5 M NaCl, followed by Milli-Q water. The biomass was resuspended in MQ water, lyophilized, weighed, and subsequently used for LPS isolation.

For intracellular replication assays in THP-1-derived macrophages, *L. pneumophila* strains were cultured in ACES-buffered yeast extract medium at 37 °C with shaking at 180 rpm until reaching OD_600nm_ = 1.8.

### 2.4. Culture of Macrophages Derived from the THP-1 Cell Line

THP-1 human monocytic leukemia cells (ATCC, TIB-202) were cultured in RPMI 1640 supplemented with 10% heat-inactivated fetal calf serum (FCS), 10 mM HEPES buffer (2-[4-(2-hydroxyethyl)-1-piperazinyl]ethanesulfonic acid), 2 mM L-glutamine, and antibiotics (100 IU/mL penicillin, 100 μg/mL streptomycin) at 37 °C in a 5% CO_2_ incubator. For subsequent assays, THP-1 cells were adjusted to 2 × 10^5^ cells/mL in RPMI 1640 with 10% FCS and seeded into 24-well culture plates (Nunc, Roskilde, Denmark). Monocyte-to-macrophage conversion was induced by adding phorbol 12-myristate 13-acetate (PMA; Sigma-Aldrich, Steinheim, Germany) to a final concentration of 50 ng/mL [17]. After PMA stimulation, non-adherent cells were removed, and the remaining adherent THP-1 cells were washed three times and then maintained for an additional 3 days in PMA-free medium, which was changed daily and contained no antibiotics. Immediately before infection assays, cells were counted to calculate and apply the appropriate infectious dose, expressed as the multiplicity of infection (MOI).

THP-1 cells differentiated according to the above procedure at a density of 2 × 10^5^ cells/mL were also cultured on round coverslips placed in 6-well adherent plates. These cultures were used to study interactions between macrophages and *L. pneumophila* strains with Fluorescence Lifetime Imaging Microscopy (FLIM) (PicoQuant GmbH, Berlin, Germany).

### 2.5. Extraction and Purification of LPS

Dried bacterial cells of strains 130b, 16D12(ΔORF8), and 16D12(ΔORF8) +pJB-ORF8 (approx. 5 g per strain) were resuspended in 100 mM phosphate buffer supplemented with 5 mM EDTA. Cell disruption was achieved using two consecutive 5 min sonication cycles. The suspension was then incubated with lysozyme (Sigma-Aldrich; 6 mg per 1 g of dry biomass) for 16 h at 4 °C. Nucleic acids were degraded by treatment with DNase and RNase (Sigma-Aldrich; 0.3 mg of enzymes per 1 g of material) at 37 °C for 2 h. This step was followed by proteolytic digestion with proteinase K (Sigma-Aldrich; 0.3 mg per 1 g of biomass) at 37 °C for 20 h. Lipopolysaccharide extraction was initiated by adding an equal volume (1:1, *v*/*v*) of 90% phenol preheated to 65–68 °C [18]. The mixture was thoroughly homogenized and incubated in a water bath at 68 °C for 2 h. Due to the pronounced hydrophobicity of the bacterial material, the entire extract was transferred directly into dialysis tubing without prior separation of the phenolic and aqueous phases. Dialysis was performed for 3 days against running tap water, followed by dialysis against Milli-Q water. The material was centrifuged at 10,000× *g* for 20 min at 4 °C. The supernatant was subsequently subjected to ultracentrifugation at 38,000× *g* for 4 h at 4 °C. The pellet was resuspended in Milli-Q water and lyophilised. Final purification of lipopolysaccharide from residual lipid contaminants was achieved by sequential washing with chloroform (10 mL) and acetone (10 mL) using a glass microfiber column. The purified material was air-dried at room temperature overnight to allow complete solvent evaporation.

### 2.6. LPS Electrodialysis

LPS (10 mg) was suspended in water with 5% sodium azide and, after transfer to dialysis tubing, electrodialysed for 48 h (500 mM; 200 V; 40 W; Bio-Rad instrument, Hercules, CA, USA) with water changes every 4 h [19]. After electrodialysis, 0.1 M TEA (triethanolamine) buffer was added to the LPS solutions, and the material was lyophilized, weighed, and destained to induce pro-inflammatory cytokines.

### 2.7. Biophysical Analysis

#### 2.7.1. Microscopy Analysis

For FLIM analysis, *L. pneumophila* strains (130b, 16D12(ΔORF8), 16D12 (ΔORF8)+pJB-ORF8, 130b+pJB1806, 16D12 (ΔORF8)+pJB1806) were prepared in MQ water (100 μL each, OD_600nm_ = 0.2) and incubated with 5 μM Syto9 fluorescent dye in 50 mM Tris buffer (pH 7.0) at 20 °C for 10 min. Following centrifugation at 5000× *g* for 10 min, the cells were washed four times with MQ water. A 30 μL bacterial suspension was applied to a macrophage-coated coverslip for immediate microscopic analysis.

Suspensions of the *L. pneumophila* strains (100 μL, OD_600nm_ = 0.2) were incubated with 0.1 µM Prodan [6-propionyl-2-dimethylaminonaphthalene] (Merck) at 20 °C for 5 min. The stained cells were centrifuged and washed four times in MQ water as described above. A 30 μL bacterial suspension was applied to a polylysine-coated coverslip for immediate microscopic analysis.

The interaction kinetics between *L. pneumophila* strains (130b, 16D12(ΔORF8), 16D12 (ΔORF8)+pJB-ORF8, 130b+pJB1806, 16D12(ΔORF8)+pJB1806) and THP-1 macrophages were analysed using FRET (Förster Resonance Energy Transfer) microscopy (MicroTime 200, PicoQuant GmbH). Syto9-labelled bacteria at an MOI of 10 were added to Nile Blue-labelled THP-1-derived macrophages cultured on round coverslips, and samples were analysed immediately.

#### 2.7.2. Steady-State Light Absorption and Emission Data

**Figure 1:**
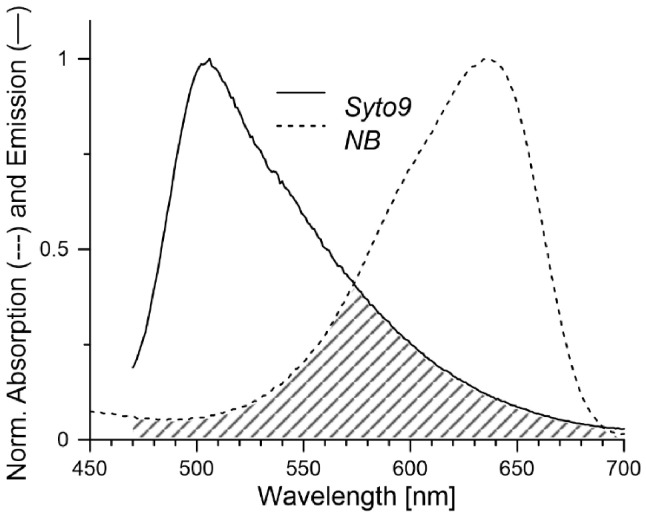
Spectral characteristics of the fluorescence labels used in nonradiative excitation energy transfer experiments. Fluorescence emission spectrum of Syto9 (solid line) and absorption spectrum of Nile Blue (dashed line). The spectra are normalized at the maximum. Marked is the spectral overlap region to determine the R_0_ value for this pair of donor-acceptor molecules.

First, the light-absorption spectra of the Syto9 and Nile Blue dyes were recorded on a Cary 50 UV–Vis spectrophotometer (Varian, Melbourne, Australia). All experiments based on steady-state fluorescence emission were performed with the application of a Cary Eclipse (Varian, Australia) spectrophotometer. The spectra revealed that the absorption maximum of Nile Blue was at 637 nm, while the emission maximum of Syto9 was at 505 nm (Figure 1. Successful FRET investigations rely on the proper selection of fluorophores for dipole–dipole interactions. In this study, Syto9 was used as an excitation energy donor fluorophore, and Nile Blue as an acceptor. The primary advantage of this donor-acceptor pair is that its distinct spectral overlap between the donor emission and the acceptor absorption spectra (marked in Figure 1). The efficiency of energy transfer is influenced by several factors, including the proximity of the donor and acceptor molecules (FRET pair), the quantum yield (*Q_D_* = 0.58) of the donor, the absorption coefficient (*ε_A_* = 18,000 M^−1^ cm^−1^) of the acceptor, the relative orientation of the FRET pair dipoles (*κ*^2^ = 2/3), and the extent of spectral overlap between the donor emission and acceptor excitation spectra. The Förster critical radius (*R*_0_) of the donor-acceptor pair was calculated using the following formula [20]:

$$R_{0}=8.79\times 10^{3}{\left[Q_{D}\kappa^{2}n^{-4}\frac{\int F_{D}\left(\lambda \right)\varepsilon_{A}\left(\lambda \right)\lambda^{4}d\lambda }{\int F_{D}\left(\lambda \right)d\lambda }\right]}^{-\frac{1}{6}}$$

where *n* is the refractive index of the medium (*n* = 1.33), *F_D_*(*λ*) is the normalized fluorescence spectrum of the donor, and *λ* is the wavelength expressed in cm. The Förster radius for pair Syto9 with Nile blue dyes was estimated as *R*_0_ = 41 Å.

#### 2.7.3. FRET Studies

The FRET efficiency for the selected samples was measured using a MicroTime 200 time-resolved fluorescence microscope (Picoquant GmbH, Berlin, Germany). The system was excited with a pulsed solid-state laser source operating at wavelengths of 470 nm and 635 nm in pulsed interleaved excitation (PIE) mode. Emission from the sample was split by a 620DCXXR dichroic mirror (AHF Analysentechnik AG, Tübingen, Germany) into two detection channels. The lifetime of the electronic excited state of the donor (*τ_DA_*) was detected using a 550/88 band-pass emission filter, while the acceptor emission was collected through a 690/70 band-pass filter (both from Chroma Inc., Bellows Falls, VT, USA). Background signals for both the donor and acceptor were evaluated separately in control experiments and subtracted from the total intensity to account for any non-specific signal. The multiexponential fluorescence lifetime of the donor in acceptor presence was analyzed using the formula expressed below:

$$\tau_{DA}=\frac{\sum_{k}A_{k}\tau_{k}}{\sum_{k}A_{k}}$$

where *A_k_* denotes the amplitude of the *k*-th exponential component.

The emitted signals were recorded with two identical SPCM-AQRH single-photon counting detectors (Excelitas Technologies, Pittsburgh, PA, USA), connected to a HydraHarp 400 time-correlated single-photon counting (TCSPC) module. The fluorescence lifetimes were quantified for both samples (donor and donor-acceptor). Data analysis was performed using the SymPhoTime 2.11 software package (PicoQuant GmbH, Berlin, Germany). The FRET efficiency (*E_FRET_*) of the system was calculated based on the fluorescence measurements using the following equation:

$$E_{FRET}=\left[1-\frac{\tau_{AvAmp}}{\tau_{D}}\right]100\%$$

The efficiency of the energy transfer had been calculated for all the image pixels and presented as a distribution on the appropriate histograms and colour-coded pictures. Finally, the distance between donor and acceptor was calculated based on:

$$R=R_{0}{\left[\frac{1}{E_{FRET}}-1\right]}^{\frac{1}{6}}$$

### 2.8. L. pneumophila Intracellular Replication Test

Bacteria at a 10 MOI dose were added to the macrophage cell culture (density 2 × 10^5^ cells/mL) and incubated at 37 °C for two hours. Subsequently, the cells were washed with 1 mL of PBS, and gentamicin (100 µg/mL) was added and incubated for 1 h at 37 °C to kill unphagocytosed bacteria. Cells were lysed with a 0.01% Triton X-100 solution and plated on BCYE medium at set time points (0, 24, 48, 72 h). The number of bacteria infecting host cells was determined by calculating the titer of bacteria released from infected cultures at 0, 24, 48, and 72 h of infection.

### 2.9. Assessment of Pro-Inflammatory Cytokine Level in THP-1-Derived Macrophages Stimulated with LPS Isolated from L. pneumophila Strains

LPS from *L. pneumophila* 130b, 16D12(ΔORF8), and 16D12(ΔORF8)+pJB-ORF8 strains was added to differentiated THP-1 cells at concentrations of 500 and 1000 ng/mL. After 4 h (for TNF-α measurement) and 24 h (for IL-6 measurement) of incubation at 37 °C, cells were centrifuged at 1500× *g* for 5 min. Supernatants were collected and stored at −80 °C until cytokine concentrations were determined. TNF-α and IL-6 concentrations in culture supernatants were quantified by sandwich enzyme-linked immunosorbent assay (ELISA) using commercial kits from Biorbyt Ltd. (Cambridge, UK). The assays were performed according to the manufacturer’s protocol. Briefly, 100 µL of culture supernatant or standard was added to antibody-coated 96-well microplates and incubated for 2 h at room temperature. After washing, 100 µL of biotinylated detection antibody was added and incubated for 1 h, followed by incubation with streptavidin–HRP conjugate for 30 min. The plates were washed, and 100 µL of TMB substrate solution was added. The reaction was stopped with the stop solution, and absorbance was measured at 450 nm using a microplate reader (VICTOR X4 Multilabel Plate Reader, Perkin Elmer, Waltham, MA, USA). Cytokine concentrations were determined from standard curves generated using recombinant cytokine standards provided in the kits. The minimum detectable concentrations were 15.6 pg/mL for TNF-α (detection range: 15.6–1000 pg/mL) and 4.69 pg/mL for IL-6 (detection range: 4.69–300 pg/mL). All samples were analyzed in technical duplicates, and all experiments were conducted in three independent biological replicates.

### 2.10. Statistical Analysis

For intracellular growth kinetics assays, bacterial counts were compared between strains at predefined time points: 0, 24, 48, and 72 h post-infection. The experimental design was treated as paired because, within each experimental run, all bacterial strains were analysed in parallel using the same batch of differentiated THP-1 macrophages, identical culture conditions, and synchronized sampling at each time point. Therefore, comparisons between strains within the same experimental run were considered matched observations.

Predefined pairwise comparisons were performed separately at each time point using two-sided paired Student’s *t*-tests. The wild-type strain 130b was compared with the mutant strain 16D12(ΔORF8), the plasmid control strain 130b+pJB1806, the mutant strain carrying the empty vector 16D12(ΔORF8)+pJB1806, and the complemented strain 16D12(ΔORF8)+pJB-ORF8. To correct for multiple testing, the Benjamini–Hochberg procedure was applied separately within each time point, treating all comparisons with the wild-type strain 130b at a given time point as a single family of hypotheses. Adjusted *p*-values ≤ 0.05 were considered statistically significant.

In addition, a predefined comparison between the mutant strain 16D12(ΔORF8) and the plasmid control strain 130b+pJB1806 was performed to determine whether the reduced intracellular proliferation of the mutant strain could be explained solely by the presence of the plasmid. This comparison was performed separately at each time point using two-sided paired Student’s *t*-tests. For this analysis, the Benjamini–Hochberg correction was applied across the four time points, and adjusted *p*-values ≤ 0.05 were considered statistically significant.

Cytokine data were analysed independently from intracellular growth kinetics data because they represented separate experimental endpoints and independent group comparisons. For cytokine measurements, comparisons between two LPS concentrations within the same LPS preparation were performed using Student’s *t*-tests. Differences between LPS preparations from different bacterial strains were assessed using one-way ANOVA followed by Tukey’s post hoc test for multiple comparisons. *p*-values ≤ 0.05 were considered statistically significant.

Intracellular growth kinetics analyses were performed in Python 3.13 using scipy version 1.16.2 and statsmodels version 0.14.5. Cytokine data were analysed using STATISTICA software, version 7.1 (StatSoft Inc., Tulsa, OK, USA).

## 3. Results

### 3.1. The N-Methyl Groups on the Acetimidoylamino GROUP in Legionaminic Acid of L. pneumophila Influence Interactions with Host Cells

To investigate the role of *N*-methylation of legionaminic acid in *L. pneumophila*, we used the wild-type strain 130b (sg1), a previously characterized mutant strain (16D12) carrying a disruption in the *orf8* gene encoding an *N*-methyltransferase responsible for legionaminic acid modification [21], and a complemented strain (16D12(ΔORF8)+pJB-ORF8) restoring *orf8* function. In addition, control strains carrying the empty vector (130b+pJB1806 and 16D12(ΔORF8)+pJB1806) were included. The 16D12(ΔORF8) mutant lacks *N*-methyl groups in legionaminic acid, providing a model to assess the functional consequences of this modification.

To determine whether *N*-methylation influences the physicochemical properties of the bacterial surface and its interaction with host cells, we performed fluorescence-based analyses, including surface polarity measurements and FLIM-FRET microscopy, as well as intracellular replication assays.

#### 3.1.1. Measurement of the Surface Polarity of *L. pneumophila* Bacteria

**Figure 2:**
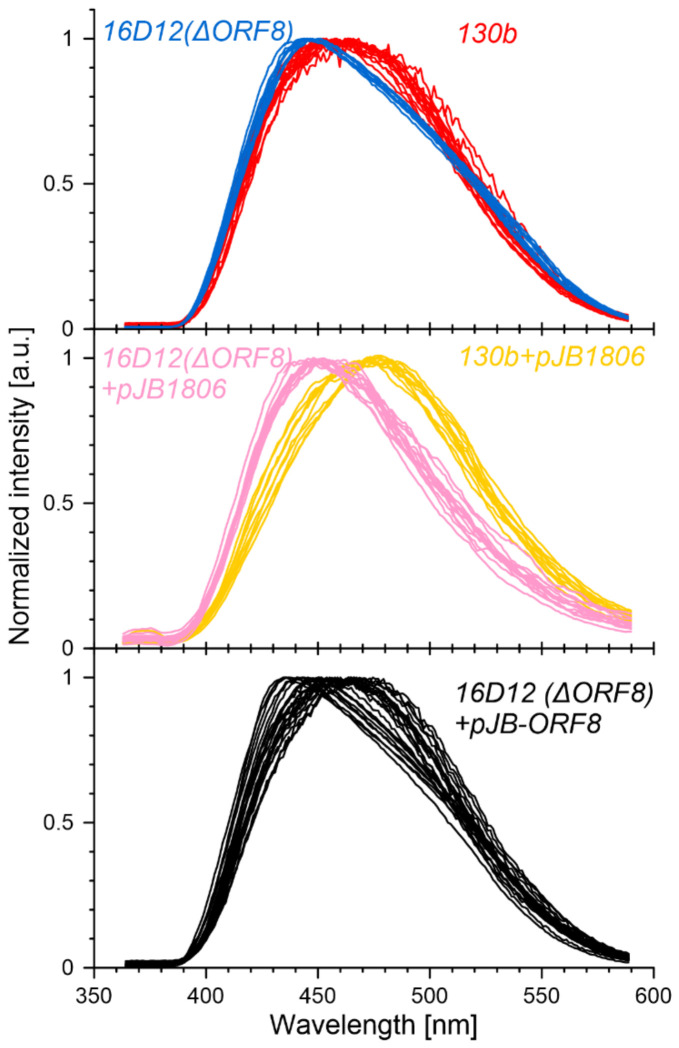
Fluorescence emission spectra of molecular label Prodan used to probe the cell surface of *L. pneumophila* strain 130b, mutant 16D12(ΔORF8), complementant 16D12(ΔORF8)+pJB-ORF8, and control strains (130b+pJB1806, 16D12(ΔORF8)+pJB1806) (marked). The spectra were normalized at the maximum. Fluorescence was recorded with excitation at 375 nm. The figure presents the results of the experiments designed to analyse possible differences in the physical properties of the cell surface of the wild-type, the mutant-type, the complementant, and control strains (130b+pJB1806, 16D12(ΔORF8+pJB1806). The results show a pronounced difference between the 16D12(ΔORF8) and 130b cells (upper panel). They also demonstrate restoration of physical properties in the complementant cells (lower panel). Fluorescence was recorded with excitation focused directly on the cell surface using a high-numerical-aperture microscope objective. Each spectrum represents the location randomly selected at the surface of a separate cell (130b and 16D12(ΔORF8), nine cells, complementant 16D12(ΔORF8)+pJB-ORF8, twenty-five cells, 130b+pJB1806, 16D12(ΔORF8)+pJB1806, ten and fourteen cells respectively).

To study possible differences in physical properties of the cell surface of *L. pneumophila* (the wild-type, the mutant-type, the complementant, and control cells), we applied staining of the cell surface with Prodan, a molecular probe sensitive to its microenvironment polarity (Figure 2). As can be seen, the fluorescence emission spectra of Prodan recorded under a microscope are distinctly different in the case of the wild-type and the mutant-type cells (multiple representative spectra are shown). The fact that the fluorescence emission spectra recorded in the case of the 16D12(ΔORF8) cells are generally shifted towards lower wavelengths (with a maximum at about 445 nm) indicates a significantly less polar environment than the fluorescence probe molecules doped into the cell membranes of the wild-type cells (130b), with a maximum at about 465 nm.

#### 3.1.2. FLIM-FRET Measurements

The FRET technique is typically used to study protein–protein interactions, but it has also been applied to research on various bacteria, including bacterial detection [22] and interactions with other molecules important from the biological standpoint [23].

To study the potential interaction of *L. pneumophila* with THP-1-derived macrophages, cells were stained with two fluorescence labels, Syto9 (bacteria) and Nile Blue (macrophage), which fulfil the conditions of effective resonance excitation energy transfer (Figure 1).

**Figure 3:**
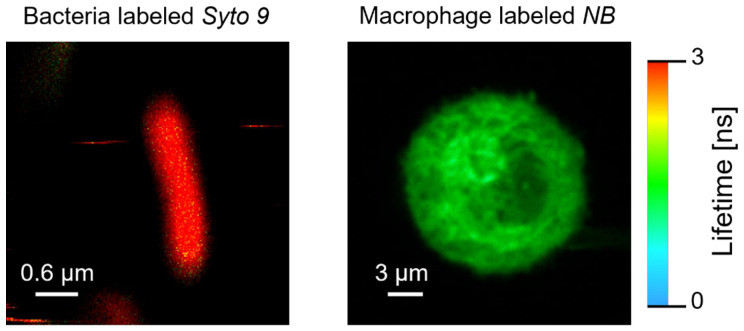
Fluorescence lifetime microscopic (FLIM) images of single cells stained with fluorescence dyes. Bacteria *L. pneumophila* strain 130b stained with Syto9 (left panel), and THP-1-derived macrophage stained with Nile Blue (right panel). The images were recorded with excitation at 470 nm and emission at 550/88 nm (bacteria) or excitation at 635 nm and emission at 690/70 nm (macrophage).

Figure 3 shows FLIM images of two types of cells examined. As can be seen, all types of cells can be effectively labelled with the fluorescence dyes selected. Moreover, distinctly different fluorescence lifetime values of Syto9 and Nile Blue and well-separated emission spectra make this pair of molecular probes suitable for precise studies of FRET and, therefore, of possible interactions (close contact) between the bacteria and the macrophage.

**Figure 4:**
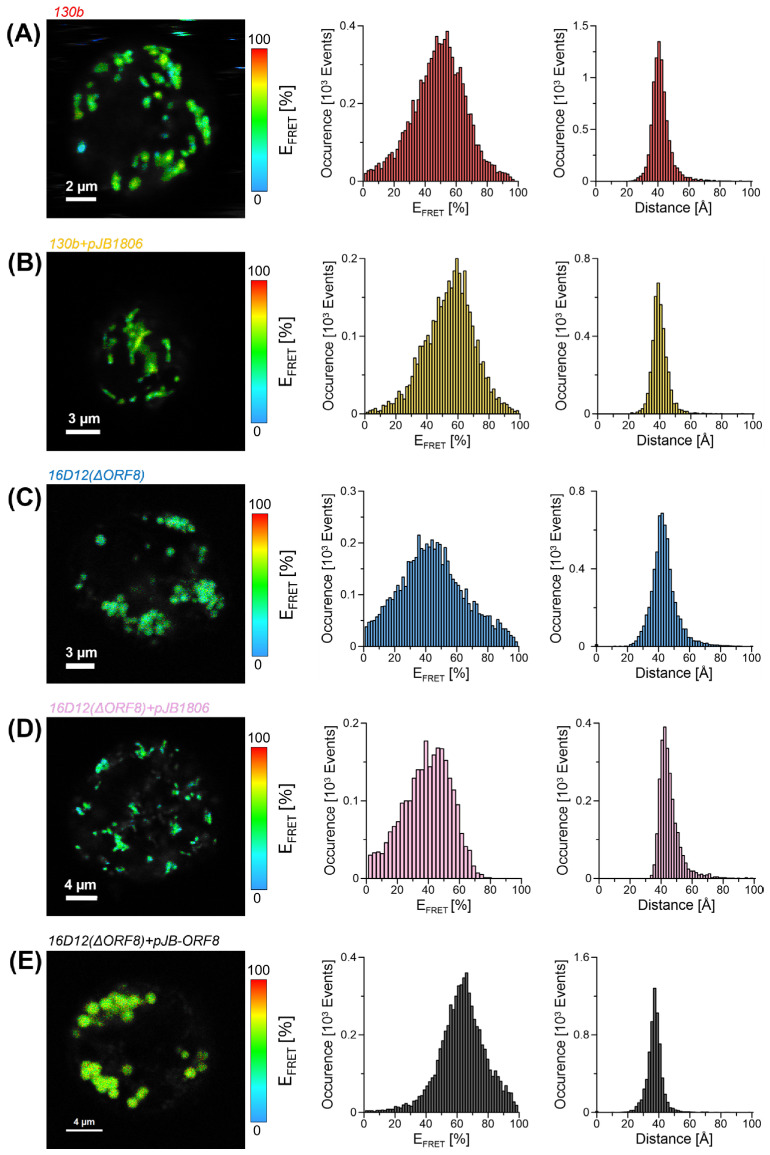
Results of analysis of the efficiency of resonance excitation energy transfer (FRET) between fluorescence dye molecules used to stain *L. pneumophila* strains (Syto9) (**A**) 130b; (**B**) 130b+pJB1806; (**C**) 16D12(ΔORF8); (**D**) 16D12(ΔORF8)+pJB1806; (**E**) 16D12(ΔORF8)+pJB-ORF8 and macrophage (Nile Blue). FRET efficiency (E_FRET_) and intermolecular distance (expressed in Å) represent interactions (close contact) of bacteria and macrophages. Note that the cell images show the exact places of interaction. For the experiments, fluorescence was excited at 470 nm (within the absorption band of Syto9) and emission was detected in two spectral channels: 550/88 nm (selective of Syto9) and 690/70 nm (selective of Nile Blue).

As can be seen from Figure 4, for the wild-type strain (*L. pneumophila* 130b, A row), a relatively broad but clearly unimodal distribution of excitation energy transfer efficiency was observed. The average maximum E_FRET_ from six independent measurements was 53%, with a standard deviation of 2 p.p. It is worth noting that the dataset shown in Figure 4 reaches a maximum E_FRET_ of 50%. This distribution corresponds to an average distance between the donor and acceptor molecules of approximately R = 40 ± 1.2 Å. These results indicate a relatively close interaction between *L. pneumophila* cells and macrophages, consistent with effective energy transfer. Although interactions can be observed in both the wild-type strain (*L. pneumophila* strain 130b, A row) and the mutant-type strain (16D12(ΔORF8), C row), the mutant-type strain exhibits weaker interactions. The excitation energy transfer efficiency for the mutant-type strain (*L. pneumophila* 16D12(ΔORF8)) had an average maximum of 39% across six independent measurements, with a standard deviation of 3 p.p. The dataset shown in Figure 4 reaches a maximum E_FRET_ of 43%. This corresponds to an average donor-acceptor distance of R = 43 ± 1.3 Å. This shift is accompanied by a broadening of the distribution and a corresponding displacement of the distance histogram towards higher values. These results indicate a reduced interaction strength in the mutant-type strain compared to the wild-type strain, consistent with weaker energy transfer efficiency. From a biophysical perspective, increased average separation between donor and acceptor molecules may result from reduced membrane apposition, altered surface organization, or decreased stability of contact sites. Consequently, the lower FRET efficiency reflects weaker or more transient interactions between the bacterial envelope and the host cell membrane. The interaction analysis between the complemented strain and macrophage across six independent measurements yielded the average maximum E_FRET_ of 57%, with a standard deviation of 3 p.p. (E row).

The wild-type strain carrying the empty vector (130b+pJB1806; B panel) exhibits E_FRET_ and distance distributions comparable to the wild-type strain, with maxima located in the 55% with a standard deviation of 2 p.p range and only minor broadening (B row).

In the case of the strain 16D12(ΔORF8)+pJB1806 (D row), the E_FRET_ distribution shifts back towards lower values (41% with a standard deviation of 1 p.p.), and the corresponding distance distribution moves towards higher R values (43 ± 1Å). The presence of empty vectors did not affect the efficiency of *L. pneumophila* interaction with macrophage cells. It has to be emphasized that the FLIM-FRET technique does not show directly molecular interactions but only indicates altered fluorophore proximity distributions. On the other hand, the fluorescence imaging of cells stained with the fluorescence probes applied for the FLIM-FRET experiments in the present work, as the excitation energy donor and acceptor (Figure 3), shows relatively homogeneous coverage of the cell surfaces. This means that the distance distribution can be applied to monitor cell–cell interactions.

#### 3.1.3. The Presence of the *N*-Methyl Group in Legionaminic Acid Modulates the Ability of *L. pneumophila* to Replicate Within Host Cells

To assess whether methyl groups bound to the acetimidoylamino group of legionaminic acid influence the intracellular replication of *L. pneumophila* in host cells, 130b, 16D12(ΔORF8), 16D12(ΔORF8)+pJB-ORF8, and control strains (130b+pJB1806, 16D12(ΔORF8)+pJB1806) were added at 10 MOI to macrophages. Unphagocytosed bacteria were eliminated with gentamicin, and at specific time points, cells were lysed and plated on BCYE medium.

**Figure 5:**
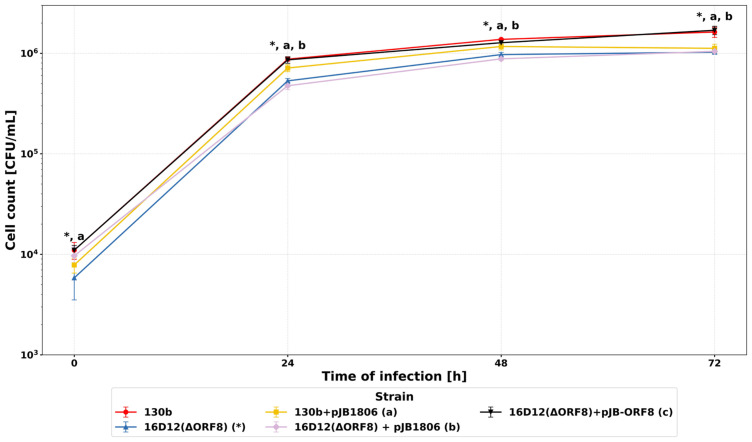
Intracellular proliferation of *L. pneumophila* strains in THP-1-derived macrophages over a 72-h time course. Strains include 130b, the 16D12(ΔORF8) mutant, plasmid-carrying strains (130b+pJB1806 and 16D12(ΔORF8)+pJB1806), and the complemented strain 16D12(ΔORF8)+pJB-ORF8. THP-1-derived macrophages were infected with *L. pneumophila* strains, and the number of viable bacteria (CFU/mL) was determined at 0, 24, 48, and 72 h. Time point 0 refers to the moment before replication but after bacterial uptake, specifically 2 h following the addition of bacteria to eukaryotic cells. Experiments were performed in three independent replicates, and error bars represent the standard deviation (S.D.). Statistical significance was assessed using a paired *t*-test with Benjamini–Hochberg correction (*p* ≤ 0.05). Symbols indicate statistically significant differences in bacterial counts relative to the wild-type strain 130b: *—16D12(ΔORF8), a—130b+pJB1806, b—16D12(ΔORF8)+pJB1806, c—16D12(ΔORF8)+pJB-ORF8.

Growth kinetics analysis in THP-1-derived macrophages demonstrated that all *Legionella* strains were able to replicate intracellularly, with the most pronounced increase, approaching nearly 2 log, observed at 24 h post-infection. At subsequent time points (48 and 72 h), the growth rate slowed down (Figure 5). The 16D12(ΔORF8) mutant was characterized by a weaker ability to multiply in macrophage cells at all time points compared to the wild-type strain, and this difference was statistically significant. The complementant strain 16D12(ΔORF8)+pJB-ORF8 showed intracellular proliferation comparable to the wild-type strain in THP-1-derived macrophages. These data indicate that *N*-methylation of legionaminic acid may contribute to efficient intracellular proliferation in THP-1 macrophages, and that loss of these groups compromises this aspect of *L. pneumophila* fitness.

A direct comparison between the 16D12(ΔORF8) mutant and the plasmid control strain (130b+pJB1806) revealed a statistically significant difference in bacterial counts at 24 and 48 h, but not at 0 and 72 h (paired *t*-test with Benjamini–Hochberg correction, *p* ≤ 0.05). This indicates that the reduced intracellular proliferation of the 16D12(ΔORF8) mutant cannot be attributed solely to the presence of the plasmid, but reflects an additional effect associated with the mutation.

#### 3.1.4. *N*-Methyl Groups of Legionaminic Acid in *L. pneumophila* LPS Influence the Level of Induced TNF-α

To assess the potential impact of *N*-methyl groups on the cytokine-inducing capacity of LPS, high concentrations (500 and 1000 ng/mL) of LPS isolated from *L. pneumophila* strains were used, as *L. pneumophila* LPS is known to be a relatively weak inducer of pro-inflammatory cytokines [24].

**Figure 6:**
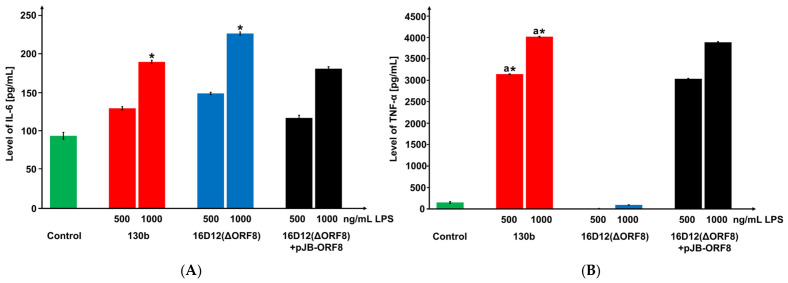
Levels of IL-6 (**A**) and TNF-α (**B**) in THP-1-derived macrophages stimulated with LPS preparations (500 and 1000 ng/mL) from *L. pneumophila* wild-type strain 130b, the 16D12(ΔORF8) mutant, and the complemented strain 16D12(ΔORF8)+pJB-ORF8. Control: IL-6.—94 pg/mL, TNF-α—142.8 pg/mL, *—statistically significant compared to the lower LPS concentration, Student’s *t*-test, *p* ≤ 0.05. a—statistically significant difference compared with the mutant-type strain 16D12(ΔORF8), *p* ≤ 0.05, Anova test, Tukey’s post hoc.

No significant differences were observed between LPS derived from the wild-type strain 130b and the 16D12(ΔORF8) mutant in their ability to induce IL-6 production in THP-1-derived macrophages. However, higher LPS concentrations from both strains significantly induced IL-6 compared to lower concentrations (Figure 6A).

In contrast, differences were evident in TNF-α induction. LPS from the wild-type strain 130b and the complemented strain 16D12(ΔORF8)+pJB-ORF8 triggered a significant increase in TNF-α production, whereas LPS from the 16D12(ΔORF8) mutant induced TNF-α at levels comparable to the control (Figure 6B).

Cytokine production in response to *L. pneumophila* LPS was dose-dependent, increasing with higher LPS concentrations. TNF-α levels were significantly higher than those of IL-6.

## 4. Discussion

Within the *L. pneumophila* species, substantial genomic and functional heterogeneity is observed among strains, promoting the selection of clones better adapted to infect the human host [12]. This epidemiological dominance is closely associated with specific structural features of LPS, which represent a key determinant of bacterial virulence and host adaptation.

Bacterial adhesion to host cells represents a critical early step in infection and is influenced by both physicochemical surface properties and specific receptor-mediated interactions. Parameters such as surface polarity, charge, and hydrophobicity govern the initial contact between bacterial and host cell membranes, while subsequent receptor-ligand interactions stabilize attachment and facilitate intracellular entry. Therefore, structural modifications of surface-exposed components, including LPS, may influence the efficiency of host–pathogen interactions.

Our previous studies demonstrated that the presence of *O*-acetyl substituents in the LPS core, as well as *O*-acetyl and *N*-methyl groups in legionaminic acid, may influence the ability of *Legionella* bacteria to interact with eukaryotic host cells [21,25,26]. The attachment of *N*-methyl groups to the 5-acetimidoylamino group in legionaminic acid of LPS of *L. pneumophila* sg1 strain 130b is mediated by an *N*-methyltransferase encoded by the *orf8* gene. *N*-methyl groups attached to the 5-acetimidoylamino moiety of legionaminic acid in *L. pneumophila* strain 130 were involved in bacterial adhesion to THP-1-derived macrophages [21]. At the early stage of these interactions, the physicochemical properties of the bacterial cell surface play a crucial role. Measurement of surface polarity, determined based on the spectroscopic properties of the fluorescence probe Prodan, revealed that the surface of the 16D12(ΔORF8) mutant was more hydrophobic than that of the wild-type strain, which may have contributed to its reduced ability to adhere to the host cell. The application of a highly sensitive fluorescence lifetime imaging microscopy (FLIM) technique, combined with Förster resonance energy transfer (FRET) analysis, enabled the determination of spatial distances between donor and acceptor fluorophore pairs. These fluorophores were used to label the wild-type strain 130b, the 16D12(ΔORF8) mutant, and macrophages, allowing for a detailed analysis of their interactions at the molecular level. A greater distance between the fluorophores labeling the 16D12(ΔORF8) mutant lacking *N*-methyl groups and macrophages may reflect weaker interactions between the cells compared to the interactions of the wild-type strain with macrophages in FRET analyses. The observed differences in E_FRET_ and donor-acceptor distance distributions should not be interpreted solely as changes in average separation, but also as indicators of the structural and dynamic organization of the bacteria-macrophage interface. Higher FRET efficiency corresponds not only to shorter distances but also to a more favourable orientation and stability of interacting components. Conversely, reduced E_FRET_ and broader distributions in the mutant strain point to increased dynamical fluctuations, reduced contact area, or altered membrane topology at the interface.

*N*-methyl groups presumably may contribute to promoting intracellular replication of strain 130b, as the 16D12(ΔORF8) mutant exhibited reduced proliferation in macrophage cells. However, studies conducted by Kooistra et al. on the wild-type *L. pneumophila* RC1 strain and the 5215 mutant lacking *N*-methyl groups of legionaminic acid residues ruled out the possibility that these groups constitute a virulence factor in the RC1 strain [27].

The presence of *N*-methyl groups in the polysaccharide part of LPS significantly contributes to its capacity to induce TNF-α. The mutant-type strain lacking these groups induced cytokine levels comparable to those of the control, indicating the importance of *N*-methylation in this response. Conversely, *N*-methyl groups do not affect LPS-induced IL-6 production, as LPS from both wild-type and mutant strains showed no significant difference in IL-6 induction. Prior studies indicate that this polysaccharide region of LPS does not activate the TLR4 receptor, as four *L. pneumophila* serogroups with distinct O-antigen structures induced comparable levels of TLR4-dependent cytokines [28,29]. While the polysaccharide O-chain seems non-essential for TLR4 activation and signalling, the oligosaccharide core sugars, including Kdo, play a critical role in this activation process [30]. Nevertheless, increasing evidence suggests that structural variations within the polysaccharide region may indirectly modulate cytokine induction by affecting the supramolecular organization, aggregation state, and accessibility of LPS to host recognition systems. The tendency of LPS to form aggregates in solution is considered an important parameter influencing the efficiency of interaction with the TLR4/MD-2 receptor complex [31,32,33]. Variations within the polysaccharide moiety, together with differences in the length and distribution of lipid A fatty acids, may alter the stability and receptor-binding properties of LPS aggregates [30]. In addition, differences in O-antigen composition and physicochemical organization of LPS have been associated with altered TNF-α in monocytes [34]. These observations are consistent with the present findings, in which the loss of *N*-methyl groups attached to the 5-acetimidoylamino group of legionaminic acid in the LPS of *L. pneumophila* sg1 selectively affected TNF-α induction without significantly altering IL-6 production.

The obtained results further suggest that *N*-methyl groups attached to the 5-acetimidoylamino group of legionaminic acid in the LPS of *L. pneumophila* sg1 may contribute to bacterial interactions with host cells, as the loss of these groups was associated with altered bacteria-host cell interactions. However, the observed effects are likely not solely attributable to the absence of *N*-methyl groups themselves but may also involve indirect changes in membrane topology and the physicochemical properties of the bacterial surface. These alterations could collectively contribute to modified host–pathogen interactions, including bacterial adhesion, intracellular proliferation, and induction of TNF-α.

## 5. Conclusions

It was demonstrated that *N*-methylation of legionaminic acid in the O-specific polysaccharide of *L. pneumophila* sg1 LPS may be associated with bacterial interactions with host cells. In contrast to earlier studies focused mainly on the structural characterization of *Legionella* LPS, the present study indicated that *N*-methyl groups may modulate the physicochemical properties of the bacterial surface, influence macrophage interactions, and contribute to intracellular replication in THP-1-derived macrophages. A novel aspect of this study was the application of FLIM-FRET analysis to investigate *L. pneumophila*-macrophage interactions. The reduced FRET efficiency observed for the mutant lacking *N*-methyl groups in legionaminic acid indicates a greater distance between fluorophores, which may reflect weaker interactions between functional groups involved in bacteria-host cell interactions. Furthermore, the results indicated that, in addition to the structure of lipid A, a well-established inducer of pro-inflammatory cytokines, specific modifications within the polysaccharide region of LPS may also help shape the inflammatory response. In particular, the presence of *N*-methyl groups in the LPS of *L. pneumophila* sg1 was associated with enhanced TNF-α induction without significantly affecting IL-6 production, suggesting a possible role for these structural modifications in the early host response to *L. pneumophila* infection.

## Data Availability

The data presented in this study are available on request from the corresponding author to protect respondent privacy.
